# Dezocine inhibits cell proliferation, migration, and invasion by targeting CRABP2 in ovarian cancer

**DOI:** 10.1515/med-2022-0541

**Published:** 2022-12-14

**Authors:** Chuanfeng Zhang, Ruirui Pan, Shuangshuang Ma, Shoucai Xu, Baosheng Wang

**Affiliations:** Department of Anesthesiology, Shandong Cancer Hospital and Institute, Shandong First Medical University and Shandong Academy of Medical Sciences, Jinan 250117, Shandong, China; Department of Anesthesiology, Shandong Cancer Hospital and Institute, Shandong First Medical University and Shandong Academy of Medical Sciences, No.440 Jiyan Road, Jinan 250117, Shandong, China

**Keywords:** ovarian cancer, dezocine, proliferation, invasion, apoptosis, CRABP2

## Abstract

Previous studies have shown that some anesthesia drugs can inhibit tumor growth and metastasis. As a clinical anesthetic drug, dezocine has been reported to play an important role in immune function. However, the effects of dezocine on ovarian cancer cell growth and metastasis are not fully understood. In this study, we found that dezocine dose-dependently inhibited the viability of ES-2 and SKOV3 cells. Dezocine suppressed the migration and invasion abilities of ovarian cancer cells, and promoted apoptosis. Moreover, the Akt/mTOR signaling pathway was also inhibited by dezocine. Furthermore, mechanism study showed that dezocine could significantly inhibit the expression of CRABP2, and CRABP2 overexpression reversed the inhibitory effects of dezocine on ovarian cancer cell proliferation and migration. In conclusion, dezocine has significant anti-tumor effects on the growth and metastatic potential of ovarian cancer cells, and CRABP2 functions as a downstream effector of dezocine.

## 1 Introduction

Ovarian cancer is the most lethal malignancy in gynecology, with high morbidity and mortality [1,2]. According to statistics, 239,000 new cases of ovarian cancer occur each year in the world, causing 152,000 deaths [3,4]. Most of the early ovarian cancer without obvious symptoms is a major cause of high mortality of ovarian cancer [5–7]. Despite significant advances in surgery and chemotherapy, the prognosis for patients with ovarian cancer is still unsatisfactory. The 5-year survival rate for patients with stage I or II ovarian cancer is 80–95%, compared with less than 30% for advanced patients [8]. Therefore, it is important to clarify the pathological mechanism of ovarian cancer and develop new treatment methods.

Anesthesia methods and drugs have been shown to affect the immune function of tumor patients, and even certain anesthetics can inhibit tumor growth and metastasis [9–11]. For example, sevoflurane has been revealed to function as an anti-tumor reagent in different types of cancer, including ovarian cancer [12–14]. As a representative opioid-receptor agonist/antagonist, dezocine has been widely used in clinical anesthesia and analgesics for postoperative cancer with minimal side effects [15–17]. Feng et al. previously reported that dezocine could regulate the secretion of IL-12 and IL-10 in dendritic cells, and enhance the activity of T cells during the maturation of dendritic cells, affecting immune function [18]. Wang et al. found that dezocine reduces the inhibitory effect of NK cells and CD4^+^ activity and the activity of CD8^+^ cells in breast cancer patients undergoing radical mastectomy, which is beneficial for the recovery of immune function [19]. These studies suggest that dezocine plays an important role in immune function. Tumor development is usually accompanied by immunodeficiency. However, whether dezocine has a direct effect on tumor cell growth and metastasis has been poorly studied.

Therefore, for the first time, we examined the effect of dezocine on the proliferation, migration, and apoptosis of ovarian cancer cells. Our data revealed an anti-tumor activity of dezocine in ovarian cancer, and identified that cellular retinoic acid binding protein 2 (CRABP2) was a downstream effector of dezocine.

## 2 Materials and methods

### 2.1 Cell culture and treatment

Human ovarian cancer cell lines ES-2 and SKOV3 were obtained from Cell Bank of Chinese Academy of Sciences (Shanghai, China). Cells were cultured in DMEM medium (Gibco, USA) supplemented with 10% FBS. Cells were treated with dezocine, and DMSO was used as negative control (NC). The cDNA sequence of CRABP2 was cloned into pcDNA3.1 vector (Sigma-Aldrich, Germany), and the blank pcDNA3.1 was used as control. The plasmids were transfected into ovarian cancer cells by Lipofectamine 2000 (Invitrogen, USA) following the manufacturer’s protocol. After transfection of CRABP2 overexpression plasmid, cells were treated with dezocine. Dactolisib (BEZ235) is a dual ATP competitive inhibitor purchased from selleck.cn. BEZ235 dissolved in DMSO was added to the medium, and the cells were treated for 48 h, then total protein was extracted and the protein expression level of CRABP2 was determined.

### 2.2 Dose-dependent assay

Cells were treated with different concentrations of dezocine (0, 2.5, 5, 10, 20, 30, 40, 50, 60, 80, 120, and 200 μg/mL) in a 96-well plate at 37°C for 24 h, respectively. Then, Cell Counting Kit-8 (CCK-8) reagent (Beijing Solarbio Science & Technology, Beijing, China) was added in each well and incubated for 90 min. The absorbance at 450 nm was recorded with a Bio-Rad microplate reader (Bio-Rad, USA).

### 2.3 CCK8 and colony formation assays

For CCK8 assay, ovarian cancer cells were exposed to dezocine in a 96-well plate at 37°C for 0, 24, 48, and 72 h, respectively. The absorbance at 450 nm was recorded according to the above steps. For colony formation assay, following treatment with dezocine for 24 h, cells were seeded into 35 mm plates with 5 × 10^2^ cells/well and cultured with DMEM at 37°C for 1–2 weeks. Colonies were fixed with 4% paraformaldehyde for 30 min and stained with 0.1% crystal violet for 30 min. The number of colonies was counted under an optical microscope.

### 2.4 Wound-healing assay

When the cells (5 × 10^5^) reached a confluency of 90% in 6-well plates, they were scraped by a sterile pipette tip, and were treated with dezocine for 0 and 24 h. Photographs were taken at the same wound location using an optical microscope (OLYMPUS, Japan), and the width of the wound was measured using ImageJ software (NIH, Bethesda, USA).

### 2.5 Transwell migration and invasion assay

Cell invasion was analyzed by Transwell chambers (8.0 μm; Millipore, MA, USA). After 24 h of treatment with dezocine or DMSO, cells (1 × 10^5^) were inoculated into the upper chamber precoated with Matrigel (BD Bioscience, CA, USA), and the lower chamber was filled with 700 µL of medium containing 20% FBS. After incubation for 24 h, cells in the upper chamber were removed, and then the invaded cells were fixed with 4% paraformaldehyde and stained with 5% crystal violet solution for 20 min. For cell migration assays, the Matrigel precoating was not performed. The migrated and invaded cells were photographed under a light microscope (100× magnification; Nikon, Japan).

### 2.6 Flow cytometry

Apoptosis was detected using the Annexin V-FITC kit (BioVision, USA) according to the manufacturer’s instructions. The cells treated with dezocine or DMSO for 24 h were collected, and incubated in serum-free medium for another 24 h. Thereafter, cells were incubated with Annexin V-FITC and PI for 30 min at room temperature in the dark. Finally, the percentage of apoptotic cells was measured using a BD FACSCalibur (Beckman Coulter, CA, USA).

### 2.7 Western blotting analysis

RIPA Lysis Buffer (CWBIO, Beijing, China) was used to extract the proteins in cells treated with dezocine for 48 h. Following which the concentration was determined by BCA kit (CWBIO), the protein samples (20 μg) were separated by 10% SDS-PAGE and transferred onto PVDF members (Millipore, Billerica, MA, USA). After being blocked with 5% dried skimmed milk for 1 h, the members were incubated with primary antibodies at 4°C overnight, and then incubated with HRP-conjugated secondary antibodies at room temperature. An enhanced chemiluminescence reagent (CWBIO) was performed to visualize the blot bands. The antibodies, including anti-Bcl-2 (Cat no. 12789-1-AP), anti-Bax (Cat no. 50599-2-Ig), anti-p-Akt (Cat no. 66444-1-Ig), anti-mTOR (Cat no. 20657-1-AP), anti-GAPDH (Cat no. 10494-1-AP), and anti-CRABP2 (Cat no. 10225-1-AP) were obtained from Proteintech Group (IL, USA); anti-cleaved Caspase 3 (Cat no. 9661), anti-Akt (Cat no. 9272), anti-p-mTOR (Cat no. 5536), anti-p70s6k (Cat no. 9204), and secondary antibodies were obtained from Cell Signaling Technology (Danvers, USA).

### 2.8 Statistical analysis

The values were presented as Mean ± SD and statistically analyzed using GraphPad Prism software. Differences between the groups were assessed using Student’s *t*-test or one-way ANOVA followed by Dunnett’s test. *P*-values less than 0.05 indicate a significant difference.

## 3 Results

### 3.1 Dezocine inhibits ovarian cancer cell proliferation and colony formation

**Figure 1:**
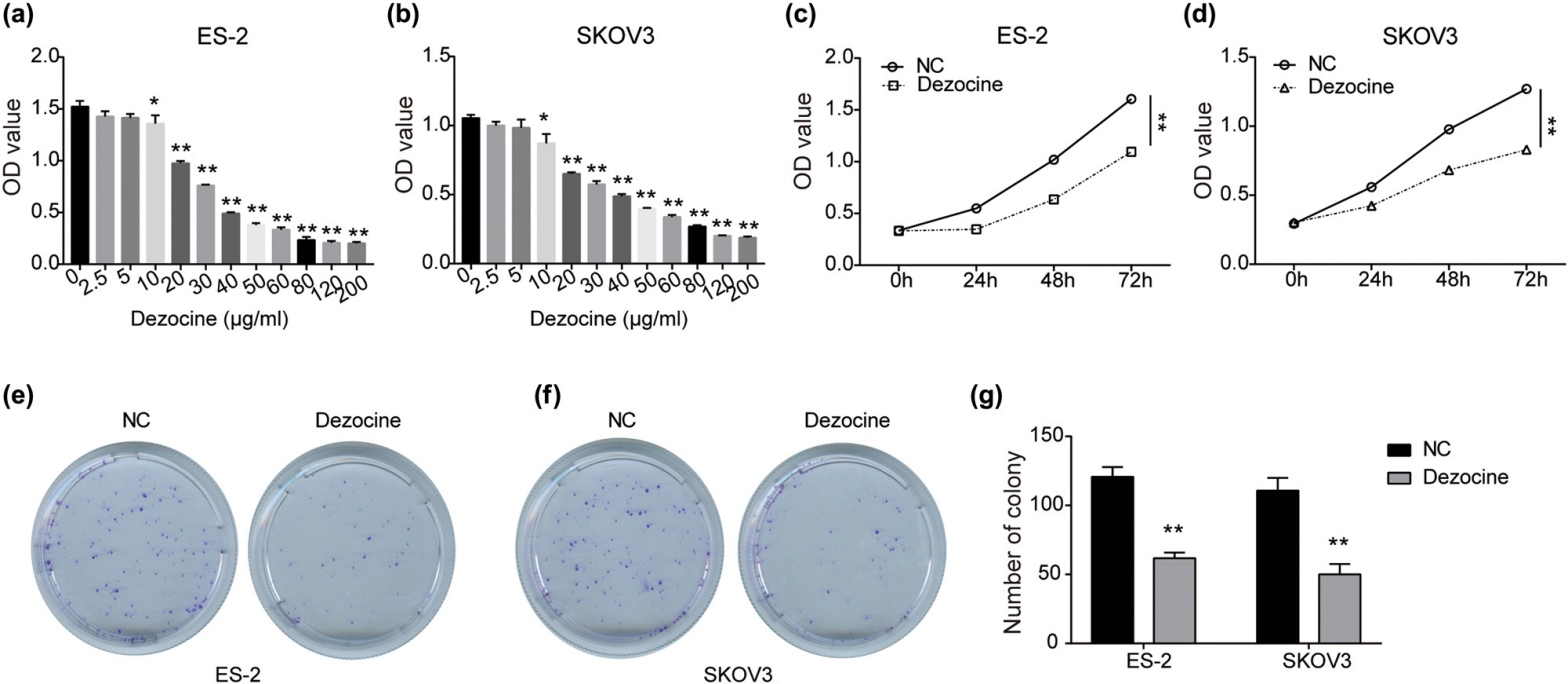
Dezocine inhibits ovarian cancer cell proliferation and colony formation. (a and b) ES-2 (a) and SKOV3 (b) cells were treated with various concentration levels of dezocine (0, 2.5, 5, 10, 20, 30, 40, 50, 60, 80, 120, and 200 μg/mL) for 24 h, and cell viability was measured by CCK8 assay. (c and d) Cell viability was measured by CCK8 assay in ES-2 (c) and SKOV3 (d) cells treated with dezocine for 0, 24, 48, and 72 h, respectively. (e and f) Colony formation assay showing inhibitory effects of dezocine on ES-2 (e) and SKOV3 (f) cells. (g) Quantitative analysis of colony formation results. ^*^ *P* < 0.05, ^**^ *P* < 0.01.

To determine the cytotoxicity of dezocine to ovarian cancer, ES-2 and SKOV3 cells were treated with different concentrations of dezocine (0, 2.5, 5, 10, 20, 30, 40, 50, 60, 80, 120, and 200 μg/mL). As shown in Figure 1a and b, the viability was significantly reduced in a dose-dependent manner after being treated with 10 μg/mL or higher concentrations of dezocine for 24 h in both ES-2 and SKOV3 cells. The IC_50_ of dezocine for ES-2 cells was 26.42 μg/mL, and for SKOV3 cells it was 29.32 μg/mL. Therefore, ES-2 cells treated with 15.85 μg/mL of dezocine was used for appropriate suppression effect in subsequent experiments, and SKOV3 cells were treated with dezocine at 17.59 μg/mL. Moreover, CCK8 assay showed that ES-2 and SKOV3 cell viability was dramatically reduced by dezocine in a time-dependent manner (Figure 1c and d). As evident from colony formation assay, compared with the NC group, the colony-forming abilities of ES-2 and SKOV3 cells were greatly inhibited after exposure to dezocine (Figure 1e–g). These results indicate the growth-inhibitory effect of dezocine on ovarian cancer.

### 3.2 Dezocine suppresses the migration and invasion of ovarian cancer cells

**Figure 2:**
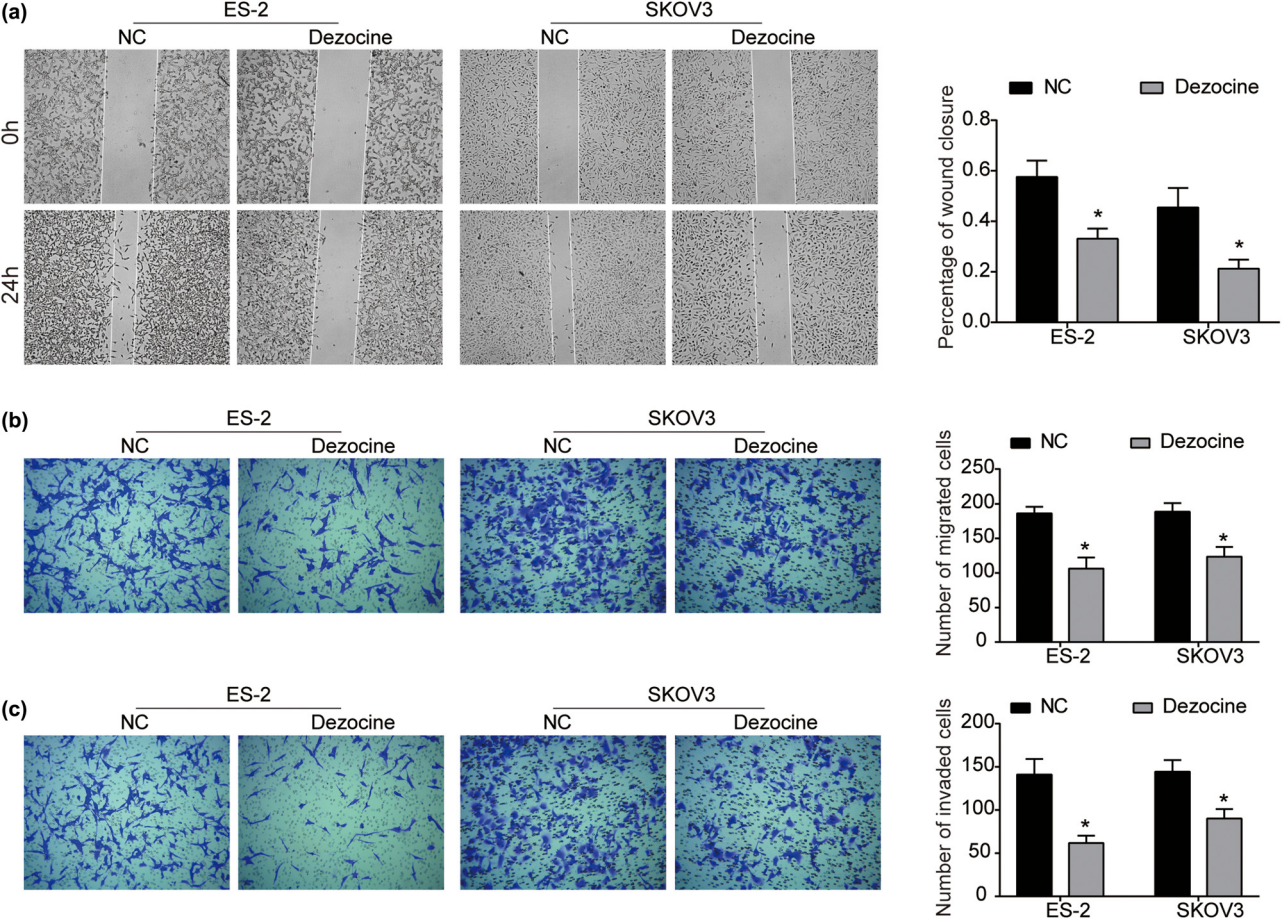
Dezocine suppresses the migration and invasion of ovarian cancer cells. (a) After cells were treated with dezocine for 0 and 24 h, wound-healing assay was performed to measure the wound closure. (b and c) Cell migration (b) and invasion (c) were measured using transwell assay in ES-2 and SKOV3 cells treated with dezocine for 24 h. ^*^ *P* < 0.05.

To determine the effect of dezocine on the mobility of ovarian cancer cells, the migration and invasion abilities of ES-2 and SKOV3 cells after exposure to dezocine were evaluated using wound-healing and transwell assays. As obvious from the wound-healing assay, compared with the corresponding control group, the migration ability of ES-2 and SKOV3 cells exposed to dezocine was significantly inhibited (Figure 2a). The migration-inhibitory effect of dezocine were further examined by transwell assay. As shown in Figure 2b, the number of migrated cells in dezocine-treated group was significantly lower than that in the control group. Further, transwell invasion assay indicated that the invasion abilities of ES-2 and SKOV3 cells were significantly repressed by dezocine (Figure 2c). Our findings reveal that dezocine possesses the activity to inhibit the metastatic potential of ovarian cancer cells.

### 3.3 Dezocine promotes apoptosis of ovarian cancer cells and inhibits the Akt/mTOR signaling pathway

**Figure 3:**
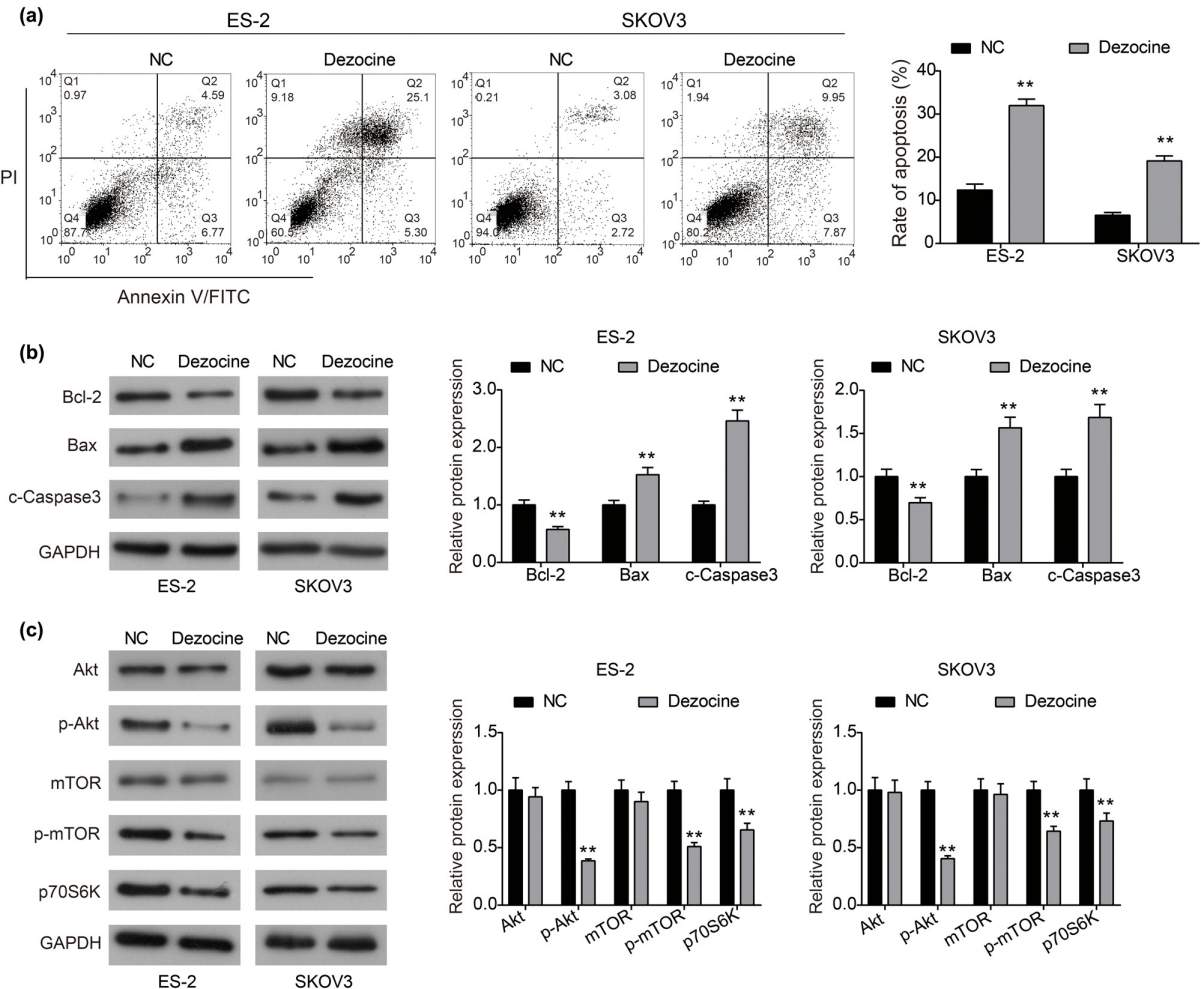
Dezocine promotes apoptosis of ovarian cancer cells and inhibits the Akt/mTOR signaling pathway. (a) Cells were treated with dezocine for 24 h, the percentage of apoptotic cells was analyzed by flow cytometry. (b) Western blot analysis of the expression of apoptosis-related proteins, Bcl-2, Bax, and cleaved Caspase 3 in ES-2 and SKOV3 cells treated with dezocine. (c) Expression of important components involved in the Akt/mTOR signaling pathway in ES-2 and SKOV3 cells, including Akt, p-Akt, mTOR, p-mTOR, and p70s6k. ^**^ *P* < 0.01.

Flow cytometry assay was used to measure the effect of dezocine on apoptosis of ovarian cancer cells. As shown in Figure 3a, dezocine significantly increased the proportion of apoptosis in ES-2 and SKOV3 cells compared to the control group. Moreover, the expression of apoptosis-related proteins was detected to investigate the molecular mechanism underlying the induced apoptosis by dezocine. As displayed in Figure 3b, the expression of anti-apoptotic protein Bcl-2 was prominently down-regulated by dezocine in both ES-2 and SKOV3 cells, while the expression of pro-apoptotic proteins Bax and cleaved Caspase 3 was prominently enhanced in dezocine-treated cells. Collectively, dezocine may induce apoptosis of ovarian cancer cells by regulating the Bcl-2/Bax axis and Caspase 3 activity.

Considering the key role of Akt/mTOR signaling pathway in regulating cell proliferation, migration, and apoptosis, we examined the effect of dezocine on the key components of the signaling pathway. As indicated by western blot analysis, we found that the total expression of Akt and mTOR was not affected by dezocine, whereas the expression of p-Akt and p-mTOR was markedly reduced in dezocine-treated group (Figure 3c). Consistently, the expression of p70S6K, an important downstream of p-mTOR, was down-regulated by dezocine in ovarian cancer cells. Therefore, dezocine may exert anti-cancer activity by inhibiting activation of the Akt/mTOR signaling pathway in ovarian cancer.

### 3.4 Dezocine inhibits the proliferation and migration of ovarian cancer by down-regulating CRABP2

**Figure 4:**
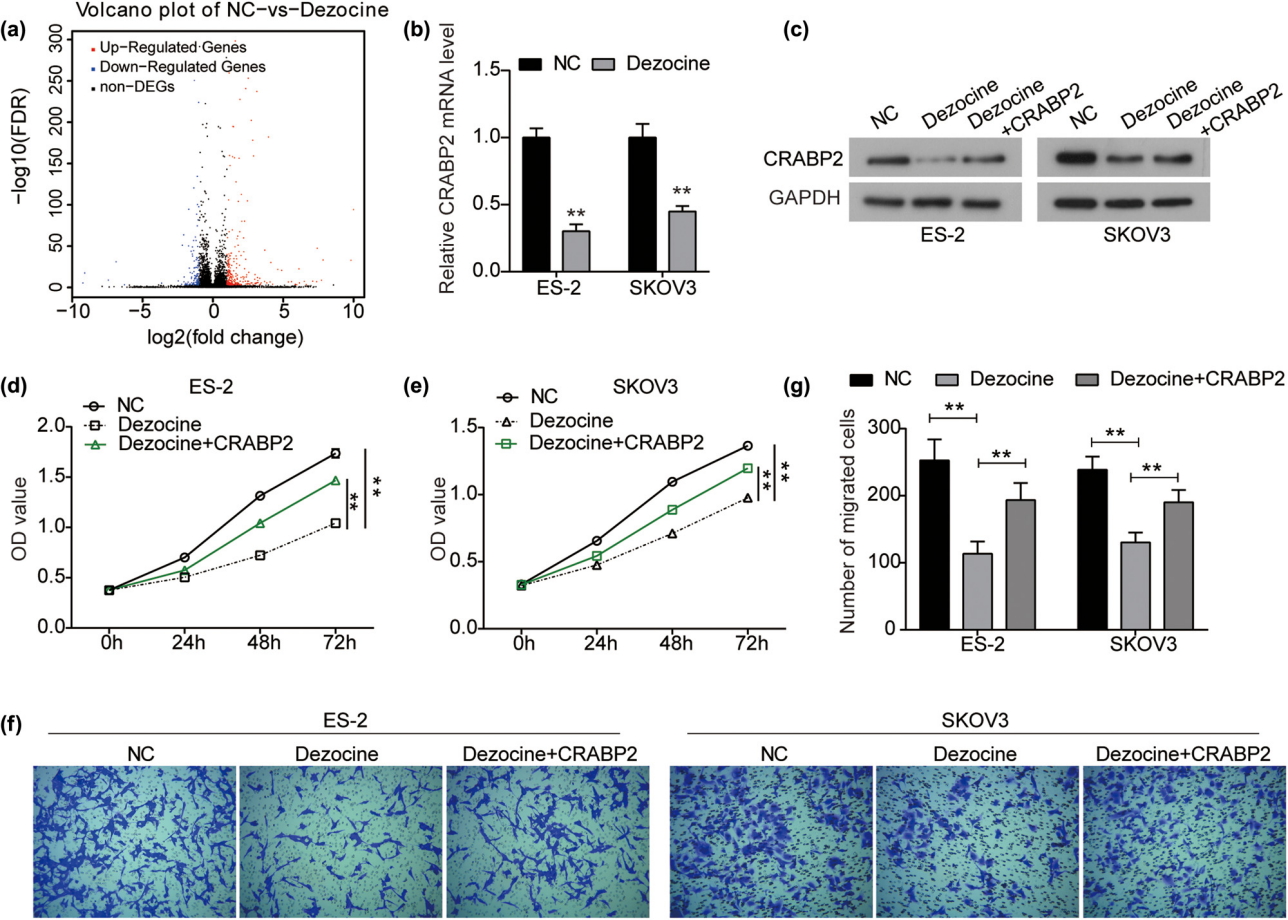
Dezocine inhibits the proliferation and migration of ovarian cancer by down-regulating CRABP2. (a) By RNA-Seq analysis, the DEGs (|log_2_ fold change| > 1 & *Q* value < 0.001) in ES-2 cells treated with dezocine. (b) Expression of CRABP2 mRNA was detected by RT-PCR analysis in ES-2 and SKOV3 cells treated with dezocine. (c) Expression of CRABP2 protein was detected by western blot analysis in ES-2 and SKOV3 cells treated with dezocine or dezocine + pcDNA3.1-CRABP2. (d and e) ES-2 (d) and SKOV3 (e) cells were treated with dezocine or dezocine + pcDNA3.1-CRABP2, and cell viability was measured by CCK8 assay. (f and g) After indicated treatment, cell migration was measured by transwell assay. ^**^ *P* < 0.01.

**Figure A1:**
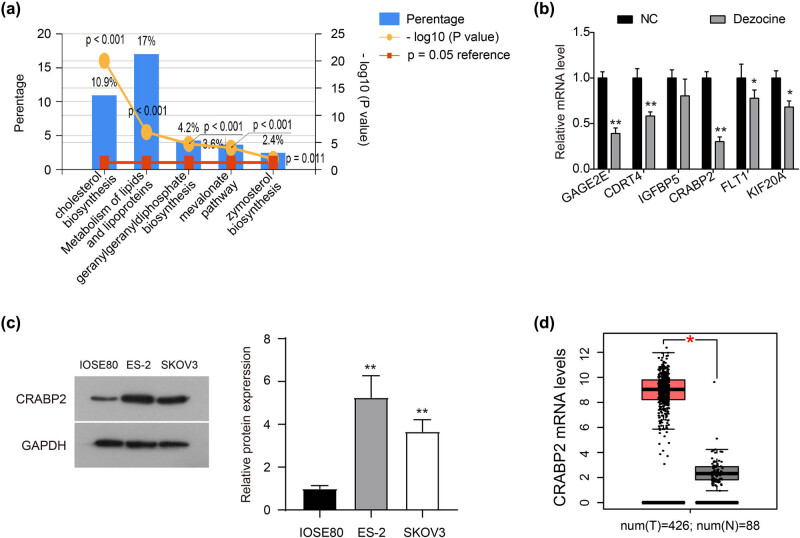
Dezocine inhibits ovarian cancer cell proliferation and colony formation. (a and b) ES-2 (a) and SKOV3 (b) cells were treated with various concentration levels of dezocine (0, 2.5, 5, 10, 20, 30, 40, 50, 60, 80, 120, and 200 μg/mL) for 24 h, and cell viability was measured by CCK8 assay. (c and d) Cell viability was measured by CCK8 assay in ES-2 (c) and SKOV3 (d) cells treated with dezocine for 0, 24, 48, and 72 h, respectively. (e and f) Colony formation assay showing inhibitory effects of dezocine on ES-2 (e) and SKOV3 (f) cells. (g) Quantitative analysis of colony formation results. ^*^ *P* < 0.05, ^**^ *P* < 0.01.

To further investigate the molecular mechanism underlying the anti-cancer activity of dezocine in ovarian cancer, RNA-Seq was performed. As shown in Figure 4a, dezocine treatment resulted in multiple differentially expressed genes (DEGs; |log_2_ fold change| > 1 & *Q* value < 0.001) in ES-2 cells, of which 259 genes were significantly up-regulated and 115 genes were down-regulated. DEGs were subjected to GO analysis using FunRich 3.1 software (Figure A1a). DEGs were mainly enriched in lipid and cholesterol metabolism and synthesis (Figure A1a).

Subsequently, we ranked DEGs according to log_2_ fold change (dezocine/NC) values, then randomly select one gene out of every seven genes, and use RT-qPCR to test the accuracy of the sequencing results. The detection results of the top 6 bits are shown in Figure A1b. High-throughput sequencing results are reliable. According to the ranking, we selected the CRABP2 with the largest log_2_ fold change (dezocine/NC) value, i.e., the CRABP2 with the largest difference in expression level, for follow-up studies.

**Figure 5:**
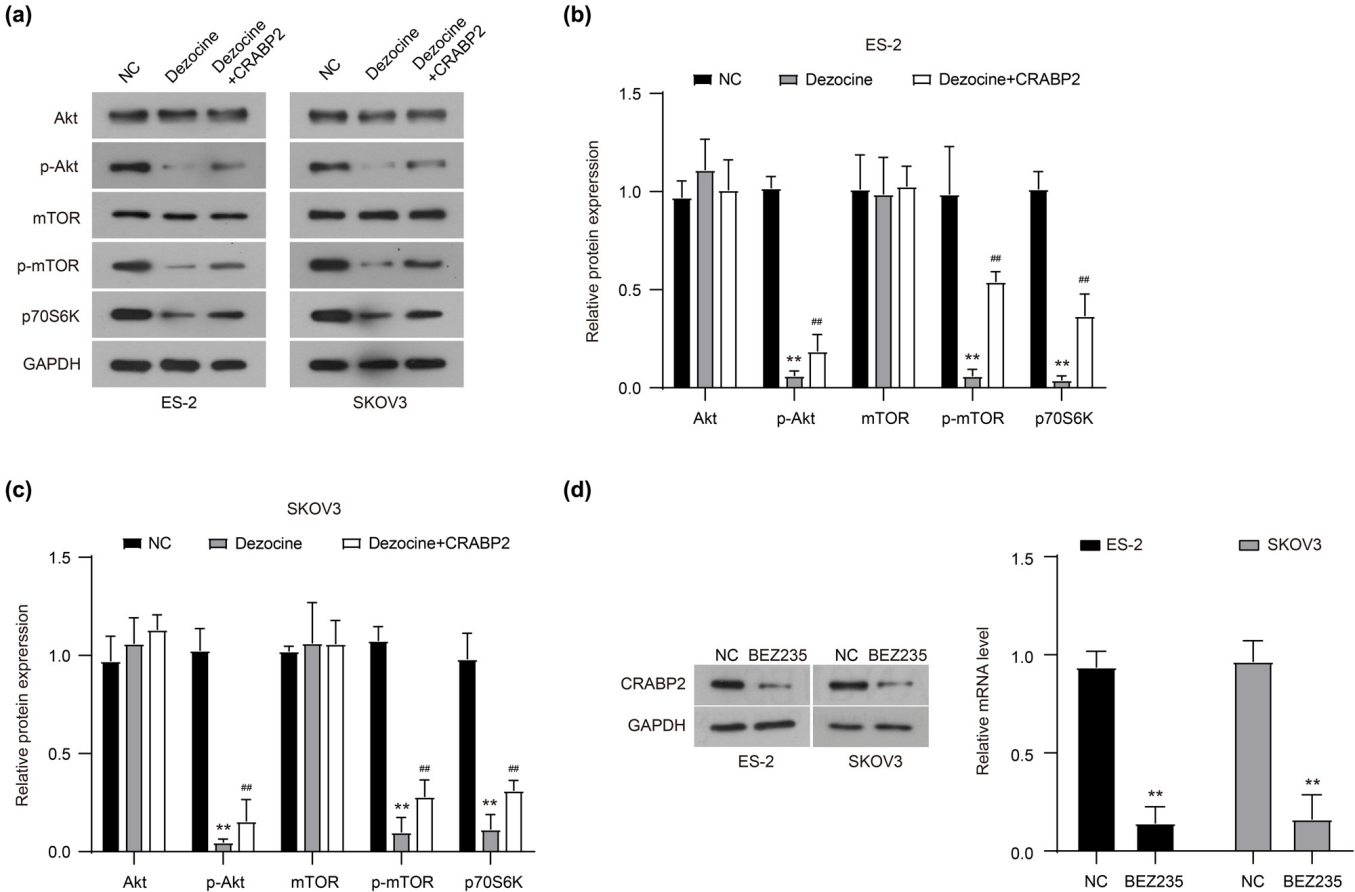
Dezocine inhibits CRABP2 expression by targeting the Akt/mTOR signaling pathway. (a–c) Expression of important components involved in the Akt/mTOR signaling pathway in ES-2 and SKOV3 cells that were treated with dezocine and CRABP2 overexpression. (d) The protein expression of CRABP2 in ES-2 and SKOV3 cells treated with BEZ235 (10 nM, a double-effect inhibitor of AKT). **P < 0.01, compared with NC group; ^##^ *P* < 0.01, compared with Dezocine group.

Similarly, there was a significant decrease in CRABP2 expression in dezocine-treated SKOV3 cells (Figure 4b). Accordingly, the expression of CRABP2 protein was significantly inhibited by dezocine in both ES-2 and SKOV3 cells, while CRABP2 overexpression could partially recover its expression (Figure 4c). Therefore, CRABP2 was a downstream effector of dezocine in ovarian cancer. Further study showed that CRABP2 overexpression reversed the inhibitory effect of dezocine on the proliferation of both ES-2 and SKOV3 cells (Figure 4d and e). Transwell assay showed that the depression in cell migration induced by dezocine was significantly rescued by CRABP2 overexpression in ovarian cancer cells (Figure 4f and g). However, CRABP2 overexpression did not rescue the inhibitory effect of dezocine on Akt and mTOR phosphorylation (Figure 5a–c). Furthermore, a dual inhibitor of AKT and mTOR phosphorylation (BEZ235, 10 nM) potently suppressed the expression of CRABP2 in ES-2 and SKOV3 cells (Figure 5d). Collectively, these results suggest that dezocine might inhibit the expression of CRABP2 by targeting the Akt/mTOR signaling pathway, and ultimately regulate the proliferation and migration of ovarian cancer cells.

## 4 Discussion

Emerging evidence has reported that dezocine could regulate immune function of cancer patients after surgery [19]. In this study, we investigated the anti-tumor activity of dezocine on ovarian cancer cells for the first time and demonstrated that dezocine could inhibit the viability of ES-2 and SKOV3 cells in a dose-independent manner. Moreover, treatment with dezocine suppressed the migration and invasion abilities of ovarian cancer cells. Additionally, dezocine was shown to induce apoptosis in ovarian cancer cells and regulate the expression of Bcl-2/Bax and cleaved Caspase 3. Taken together, our data indicate that dezocine functions as a potential anti-tumor reagent in the therapy of ovarian cancer.

It is generally known that the Akt/mTOR signaling pathway, as a classic signaling pathway, is frequently activated in tumors and plays a key role in the malignant transformation of tumors, including ovarian cancer [20,21]. Overactivation of the Akt/mTOR pathway is closely associated with tumorigenesis, tumor progression, and drug resistance [22,23]. Therefore, inhibiting activation of this signaling pathway is regarded as a promising method to control the growth and metastatic ability of tumors [24,25]. Many antitumor drugs could inhibit the activation of this signaling pathway [26,27]. In the present study, our data demonstrated that the expression of p-Akt and p-mTOR was markedly reduced with dezocine treatment, suggesting that dezocine could suppress activation of the Akt/mTOR signaling pathway in ovarian cancer cells. Taken together, these data indicate that the anti-tumor activity of dezocine may be associated with the inhibition of the Akt/mTOR signaling pathway.

CRABP2, a member of the intracellular lipid-binding protein family, has been showed to transport RA to the retinoic acid receptor in the nucleus [28,29]. Increasing study has reported that dysregulated CRABP2 is associated with the progression of tumors [30]. Our data also demonstrated that CRABP2 is highly expressed in ovarian cancer cell lines and tissues (Figure A1c and d). Furthermore, CRABP2 is highly expressed in pancreatic ductal adenocarcinoma [30], breast cancer [31], and non-small cell lung cancer (NSCLC) [32], its high expression is associated with poor prognosis of patients with NSCLC or ER-negative breast cancer [32,33]. Yu et al. report that CRABP2 promotes the invasion of pancreatic cancer cells through stabilizing the interleukin 8 expression [34]. Wu et al. demonstrate that CRABP2 enhances lung cancer metastasis by HuR and integrin β1/FAK/ERK signaling [35]. These results highlight the oncogenic role of CRABP2 in the progression of cancers. However, the role of CRABP2 in breast cancer growth and metastasis has been interpreted differently. In ER + breast cancer cells, CRABP2 suppresses tumor metastasis by inhibiting ubiquitination of Lats1; while CRABP2 enhances ubiquitination of Lats1 and promotes tumor metastasis [31]. Toyama et al. show that CRABP2 is strongly expressed in serous carcinoma, which is identified as a candidate subtype-specific biomarker for ovarian cancers [36]. In this study, we found that CRABP2 was markedly inhibited by dezocine in ovarian cancer cells at both mRNA and protein levels by inhibiting the Akt/mTOR signaling pathway activation. Moreover, CRABP2 overexpression could partially reverse the inhibitory effect of dezocine on cell proliferation and migration. Taken together, CRABP2 might exert an oncogenic role in ovarian cancer and function as a downstream effector of dezocine.

In the present study, our findings identified the anti-cancer activity of dezocine in ovarian cancer. Dezocine could inhibit cell proliferation and invasion, and promote apoptosis. Moreover, mechanism study suggested that CRABP2 was a downstream effector of dezocine, which is involved in the anti-cancer activity of dezocine. Dezocine may represent a promising novel targeted agent in ovarian cancer.
